# Effect of blood protein concentrations on drug-dosing regimes: practical guidance

**DOI:** 10.1186/1742-4682-10-20

**Published:** 2013-03-18

**Authors:** Konstantin G Gurevich

**Affiliations:** 1 Moscow State University of Medicine and Dentistry, Russian Federation, Delegatsakaya street 20/1, Room 216, Moscow 127473, Russia

**Keywords:** Pharmacokinetics, Blood protein, Drug dosing regime

## Abstract

In this article the importance of blood proteins for drug dosing regimes is discussed. A simple mathematical model is presented for estimating recommended drug doses when the concentration of blood proteins is decreased. Practical guidance for drug dosing regimes is discussed and given in the form of a figure. It is demonstrated that correction of drug dosing regimes is needed only for when there is a high level of drug conjugation with blood proteins and a high degree of hypoalbuminaemia. An example of the use of this model is given.

## Background

Blood proteins are transporters for most drugs. The free drug fraction has therapeutic activity, but on the other hand it has potentially toxic side effects. Conjugation of a drug with blood proteins is not constant and can be change in many clinical situations, the most common of which is hypoalbuminaemia [1]. The concentrations of blood proteins that bind drugs strongly has been shown to be important. When these concentrations change, clinicians need to adapt their dosing regimens rationally, on the basis of the changed pharmacokinetic characteristics of the drug. For example, blood proteins play critical roles in antibacterial drug dosing regimes for terminal patients [2-5]. Among such patients, hypoalbuminaemia is very common, with reported incidences as high as 40–50% [4,6].

It has been demonstrated that changes in protein binding can affect antiviral activity and patient management. A change in protein binding causes a clinically important change in the relationship between total and unconjugated concentrations of the drug. Thus, blood proteins have critical effects on individual drug doses regimes and the efficacy of antiviral therapy for HIV-infected patients [3,7-10].

Blood proteins also influence the penetration of drugs into tissues [11-13] and drug elimination and metabolism [14,15]. Changes of free drug concentrations due to hypoalbuminaemia can dramatically influence the therapeutic effects and induce side-effects [16]. For example, proteins reduce the toxicity of anti-tumor drugs [17].

In obese patients, blood protein concentrations might be the main factor influencing the drug dosing regimes [18,19]. The same mechanisms have been discussed for pregnancy [20,21] and for sex differences in drug dosing [22,23]. We previously demonstrated critical changes of character in the kinetics of drug effects due to blood proteins [8,24,25].

Unfortunately, recommendations for drug dosing regimes in cases in which blood protein concentrations are decreased are unclear. In this article we try to provide practical guidance for drug dosing in relation to blood protein concentration.

## Results

As is well established, drugs in the blood can be found in both free and conjugates (bound) forms [26]. Conjugation occurs with blood proteins, mainly albumin, and is reversible:

(1) $$\underset{\mathrm{free}\;\mathrm{drug}\;\mathrm{fraction}}{\underbrace{\mathrm{Drug}}}+\mathrm{Blood}\;\mathrm{protein}↔\underset{\mathrm{conjugate}\;\mathrm{drug}\;\mathrm{fraction}}{\underbrace{\mathrm{Complex}\;\mathrm{drug}-\mathrm{blood}\;\mathrm{protein}}}$$

From equation (1), the degree of drug conjugation (DC) can be calculated as:

(2) $$\mathrm{DC}=\frac{\mathrm{free}\;\mathrm{drug}\;\mathrm{fraction}}{\mathrm{total}\;\mathrm{drug}\;\mathrm{in}\;\mathrm{blood}\;\left(\mathrm{free}+\mathrm{conjugated}\;\mathrm{fractions}\right)}\cdot 100\%$$

The higher the DC, the less of the drug is free in the blood, and the lower its therapeutic effect (the conjugated drug cannot interact with its molecular targets, i.e. receptors; a conjugated drug is only a “reserve” of that drug).

**Figure 1:**
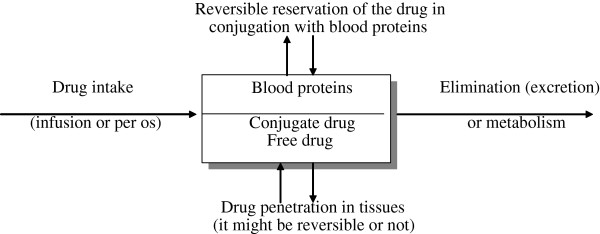
Influence of drug-binding proteins on pharmacokinetics of drugs.

The interaction of a drug with blood proteins determines its pharmacological activity and its accumulation in tissues. Only the free fraction can penetrate into tissues from the blood, and only the free fraction of the drug is extracted or metabolized [1,26]. A scheme showing the influence of blood proteins on drug pharmacokinetics is given in Figure 1.

**Figure 2:**
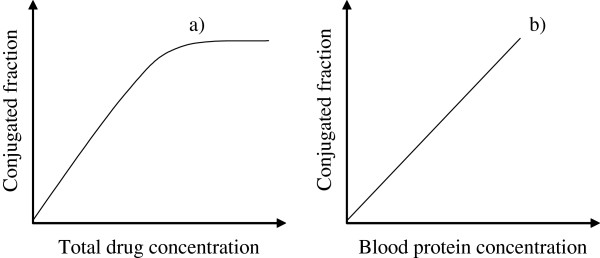
Concentration of conjugated drug fraction: influence of total drug concentration (a), and influence of blood protein concentration (b).

Only a few drugs have specific blood-transport proteins; for most, the main binding protein in the blood is albumin. Drug conjugation with blood proteins (i.e. albumin) is determined by several factors [27]:

1. *The chemical structure of the drug*.

2. *Possible interaction with blood proteins for several drugs or their metabolites.*

3. *Possible interactions with blood proteins for the drug and some endogenous substances.*

4. *Total drug concentration in the blood*. Because the capacity of proteins for a drug is limited, it can be shown that for high drug concentrations, the DC decreases, i.e. the amount of free drug increases (Figure 2a). In this situation the drug’s side effects are more likely to be manifested.

5. *Concentration of blood proteins*. Increasing the concentration of blood proteins lowers the DC, i.e. decreases the free drug concentration (Figure 2b). On the other hand, a decrease of blood proteins can influence the drug’s side-effects [1]. In this situation, correction of drug dosage is needed.

**Figure 3:**
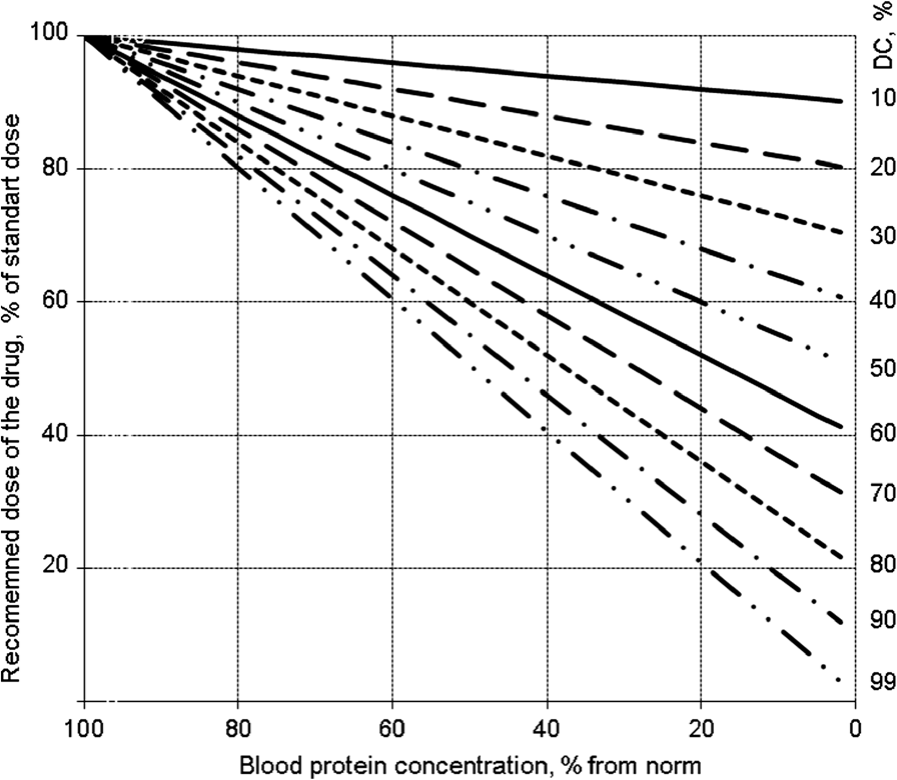
**Recommended drug dosing regimes when blood protein concentration is decreased (equation (13)).** Numbers near lines are DC. To find the recommended dose, construct a perpendicular from the X axis (point of blood protein concentration) until it intersects the line of DC for the current drug. The Y axis value at this point gives the recommended drug dose.

We offer a simple model estimation for the case of decreased blood protein concentration (see below). From our point of view, the model results make clinical sense. The model estimation for equation (13) is given in Figure 3. It is follows that for drugs with high DC, the dosing regimes have to be altered when the blood protein concentration is decreased. For drugs with low DC, this is not so important.

Therefore, in clinical practice, it is first necessary to measure the blood protein concentrations as percentages of normal values. Then, from pharmacological databases, the doctor should note the DC for the drug needed for the patient. For example, the DC for cefixime is about 70% [4,5]. Using Figure 3, it can easily be found that when the total blood protein concentration is 80% of normal, 88% of the usual dose of the drug has to be used. When the blood protein concentration is 40% of normal, 58% of usual dose of the drug is required. Thus, the change in drug dosage is uncritical in the first case but critical in the second.

## Discussion

Blood proteins play a critical role in the dosing regimes and side-effects of drugs [4,5,16]. In cases of hypoalbuminaemia the profile of therapeutic safety of drugs becomes lower, and probability that drug side-effects will develop is increased [1].

In this article we have demonstrated that the free fraction of the drug is dramatically changed in hypoalbuminaemia only for drugs with high DC values, i.e. some antibacterial and antiviral drugs. Because the free drug concentration directly influences the drug’s therapeutic and side effects [16,17], dose regimes only have to be corrected for drugs with high DC values in hypoalbuminaemia cases.

We present a simple model that allows the same concentration of free drug to be attained in cases of both normal and decreased blood protein concentration. The model gives practical guidance for drug dosing regimes in hypoalbuminaemia (Figure 3). It is ready for clinical use and needs no additional calculations. Physicians need to know only two things before using the model: the DC of the drug and the percentage decrease of blood protein concentration below normal values. The first is given in drug annotations, the second can be easily measured in a clinical laboratory.

For patients with bleeding, certain solutions (i.e. detraining) are used to restore the blood volume. It has to be noted that such solutions contain no blood proteins. Therefore, these patients require additional treatment with albumin or whole blood, or the drug dosing regimens have to be changed.

It has to be remarked that our model has important limitations:

1. The results are true only for drugs with linear dependence of blood concentration on intake dose. This seems valid for any intravenous or intramuscular drug intake. But for oral intake, the blood drug concentration depends not on only on the dose but also on food intake, drug solubility, gastric and intestinal pH, and numerous other factors. However, hypoalbuminaemia usually means intensive care is needed, so most drugs need to be given intravenously or intramuscularly.

2. The model takes no account of possible changes in the equilibrium constant, *K*. These might correspond to blood pH changes, changes of blood temperature (due to changes of total body temperature), genetic or chemical modifications of blood proteins, or other factors.

3. The results are valid only for drugs binding to one type of blood protein transporter (or several transporters with similar values of *K*). However, for antimicrobial and antiviral drugs, this assumption is true, so our model seems to be clinical relevant.

### Model estimation

Let us calculate safe drug dosing regimes when the blood protein concentration is reduced. It has to be remarked that such a decrease in blood proteins is a common clinical situation: it occurs in renal and liver diseases, bleeding and so on. From our point of view, the correction of drug dosing regimes in such cases is most useful for drugs with high DC values.

In the simplest situation there is only one drug (*L*) and one type of blood protein (*P)*. Their interaction is reversible with equilibrium constant (*K*):

(3) $$L+P\overset{K}{↔}\mathrm{LP}$$

where *LP* is the complex of drug with blood protein (i.e. conjugated drug).

Here, the equilibrium constant can be calculated as (square brackets are used to denote concentrations of appropriate substances):

(4) $$K=\frac{\left[L\right]\cdot \left[P\right]}{\left[\mathrm{LP}\right]}$$

and DC is:

(5) $$\mathrm{DC}=\frac{\left[\mathrm{LP}\right]}{\left[L_{0}\right]}$$

where [*L*_0_] is total drug concentration in the blood. Or:

(6) $$\left[\mathrm{LP}\right]=\mathrm{DC}\left[L_{0}\right]$$

It is obvious that:

(7) $$\left[L_{0}\right]=\left[\mathrm{LP}\right]+\left[L\right]$$

So, given (7), equation (4) can be rewritten as:

(8) $$K=\frac{\left[L_{0}\right]\left(1-\mathrm{DC}\right)\left[P\right]}{\mathrm{DC}\left[L_{0}\right]}=\frac{\left(1-\mathrm{DC}\right)\left[P\right]}{\mathrm{DC}}$$

or:

(9) $$\mathrm{DC}=\frac{\left[P\right]}{K+\left[P\right]}$$

If the blood protein concentration decreases *а-*fold, equation (9) can be rewritten as:

(10) $$DC_{1}=\frac{a\left[P\right]}{K+a\left[P\right]}$$

where *DC*_1_ is the DC when the blood protein concentration is decreased.

From equations (6) and (9), the free drug concentration in normal blood is:

$$\left[L\right]=\left[L_{0}\right]\left(1-\mathrm{DC}\right)$$

and when the blood protein concentration is decreased:

$$\left[L_{1}\right]=\left[L_{01}\right]\left(1-DC_{1}\right)$$

where [*L*_01_] is the total drug concentration in such a case.

It can be assumed that when [*L*] = [*L*_1_] there will be no side effects corresponding to the increased free drug fraction. I.e.:

(11) $$\left[L_{01}\right]\left(1-DC_{1}\right)=\left[L_{0}\right]\left(1-\mathrm{DC}\right)$$

From equations (9), (10), equation (11) can be rewritten as:

(12) $$\frac{\left[L_{01}\right]}{\left[L_{0}\right]}=\frac{1-\mathrm{DC}}{1-DC_{1}}=\frac{1-\mathrm{DC}}{1-\frac{a\left[P\right]}{K+a\left[P\right]}}$$

Or:

(13) $$\frac{\left[L_{01}\right]}{\left[L_{0}\right]}=\frac{1-\mathrm{DC}}{1-\frac{\mathrm{aDC}\left[P\right]}{\left(1-\mathrm{DC}\right)\left[P\right]+\mathrm{aDC}\left[P\right]}}=\frac{1-\mathrm{DC}}{1-\frac{\mathrm{aDC}}{1-\mathrm{DC}+\mathrm{aDC}}}$$

Equation (13) allows changes in [*L*_01_] to be calculated and compared with [*L*_0_], with the limitation [*L*] = [*L*_1_]. Graphically, the results of estimation are illustrated in Figure 3. These calculations are valid when the blood concentration of the drug is a linear function of its dose.

## Competing interest

The author declares that he has no competing interest.
